# BIPV-Powered Smart Windows Utilizing Photovoltaic and Electrochromic Devices

**DOI:** 10.3390/s120100359

**Published:** 2011-12-30

**Authors:** Rong-Hua Ma, Yu-Chia Chen

**Affiliations:** 1 Department of Mechanical Engineering, ROC Military Academy, Kaohsiung 830, Taiwan; 2 Institute of Materials Engineering, National Pingtung University of Science and Technology, Pingtung 912, Taiwan; E-Mail: e76512000@yahoo.com.tw

**Keywords:** absorptance, BIPV, electrochromic, tungsten oxide (WO_3_)

## Abstract

A BIPV-powered smart window comprising a building-integrated photovoltaic (BIPV) panel and an all-solid-state electrochromic (EC) stack is proposed. In the proposed device, the output voltage of the BIPV panel varies in accordance with the intensity of the incident light and is modulated in such a way as to generate the EC stack voltage required to maintain the indoor illuminance within a specified range. Two different EC stacks are fabricated and characterized, namely one stack comprising ITO/WO_3_/Ta_2_O_5_/ITO and one stack comprising ITO/WO_3_/lithium-polymer electrolyte/ITO. It is shown that of the two stacks, the ITO/WO_3_/lithium-polymer electrolyte/ITO stack has a larger absorptance (*i.e*., approximately 99% at a driving voltage of 3.5 V). The experimental results show that the smart window incorporating an ITO/WO_3_/lithium-polymer electrolyte/ITO stack with an electrolyte thickness of 1.0 μm provides an indoor illuminance range of 750–1,500 Lux under typical summertime conditions in Taiwan.

## 1. Introduction

Many transition metal oxides have been investigated regarding their potential for use in electrochromic applications [1–16]. Among these oxides, tungsten oxide (WO_3_) is the most commonly used inorganic electrochromic compound due to its many favorable properties, including a high capacity for reversible Li^+^ insertion, a high coloration efficiency, good reversibility, relatively low price, and non-toxicity [3].

Smart windows, which change from transparent to frosted to opaque with the simple turn of a switch, provide a promising solution for controlling the amount of heat or light entering a building, and are invaluable in reducing heating, air-conditioning and lighting costs. Such windows generally incorporate an electrochromic (EC) stack comprising an active EC layer and a passive counter electrode sandwiched between two transparent electrical conductors (e.g., tin-doped indium oxide, ITO). Typically, the active EC layer consists of a thin glass plate coated with a thin film of WO_3_, NiO [4,5], V_2_O_5_ or MoO_3_ [6], while the counter electrode comprises a thin glass plate coated with lithium electrolyte [7] or some other form of anodically-colored transition metal oxide [8].

Ho *et al.* [1] presented a solid-state EC system comprising a WO_3_/Prussian blue (PB) thin film coupled with a proton-conducting, solid polymer electrolyte. It was shown that the device could be switched between a darkened state and a bleached state by applying electrical voltages of +1.2 V and −0.6 V, respectively. In the same year, Zhang *et al.* [2] proposed a smart window with a WO_3_/lithium-polymer electrolyte/V_2_O_5_ structure and showed that the transmittance of the device reduced from 74% to 12% within 60 s of switching the applied voltage from 1.8 V to −1.2 V. Later, Özer [3] used a sol-gel spin coating technique to coat glass slides with thin WO_3_ films prepared from a solution of tungsten oxychloride and tungsten complex precursor. The experimental results showed that the optical and electrochemical properties of the WO_3_ films exhibited electrochemical reversibility beyond 1,600 cycles with no change in performance. Maccari *et al.* [9] developed medium size (300 × 300 mm^2^) EC devices for architectural and automotive applications comprising WO_3_/vanadium pentoxide as the active electrode and a lithium-coated glass plate as the counter electrode.

Various deposition methods have been proposed for the preparation of thin WO_3_ films, including vacuum evaporation, electrochemical deposition, sol-gel deposition, chemical vapor deposition, spray pyrolysis, and sputtering [7,17–20]. Among these methods, magnetron sputtering has emerged to be of potential interest for production of wide-spread products due to its many favorable properties [7]. Kamal *et al.* [17] used an RF-sputtering technique to deposit amorphous WO_3_ films on glass substrates and found that the coloration of WO_3_ under the application of an external electrical field is the result of a hopping of the polarons within the film. Sivakumar *et al.* [18] deposited thin WO_3_ films on fluorine-doped tin oxide (FTO) coated glass slides using an electron beam evaporation technique with different substrate temperatures. The experimental results showed that the films deposited using a higher substrate temperature yielded a smaller modulation of the visible spectrum than those deposited at a lower temperature. Zhang *et al.* [20] fabricated self-organized macroporous WO_3_ films on ITO-coated glass slides via the anodic oxidation of DC-sputtered W layers. The anodized WO_3_ films were shown to have both a rapid switching speed (*i.e*., 8 s) and a large color contrast (*i.e*., 50%).

Smart windows are an effective means of reducing energy costs and achieving adjustable lighting levels for user comfort [21]. They vary the throughput of radiant energy and visible light by means of glass plates coated with a thin film having photochromic, thermochromic or electrochromic properties [22]. Lampert [23] examined various electrically-activated chromogenic glazings containing dispersed liquid crystals, dispersed particles and electrochromics, respectively. The results showed that transmittance ranges of 20–60%, 10–50% and 0.1–10% could be obtained with switching speeds of 100–200 ms given applied voltages ranging from 0–20 V to 150 V or more. Baloukas *et al.* [24] developed a smart window in which the single WO_3_ active layer within conventional devices was replaced by an EC interference filter comprising a stack of dense and porous WO_3_ layers. It was shown that the dense overlaying coating had no effect on the coloration of the underlying porous coating and a voltage switch of −0.6 V to +1.5 V was sufficient to prompt a change from a bleached state to a darkened state.

Building-integrated photovoltaics (BIPV) are finding increasing use in the construction of new buildings as either a primary or auxiliary source of electrical power. In the present study, a BIPV module is integrated with an EC stack in order to realize a BIPV-powered smart window. In the proposed device, the variable output voltage from the BIPV module is used to drive the EC stack and is modulated in such a way as to achieve the specified indoor illumination conditions. Two EC stacks are considered, namely one stack with an ITO/WO_3_/Ta_2_O_5_/ITO structure and a second stack with an ITO/WO_3_/lithium-polymer electrolyte/ITO structure. The diffraction pattern of the WO_3_ thin-film deposited on the ITO glass slide in each stack is examined using an X-Ray Diffraction (XRD) technique. The coloration and bleaching properties of the two stacks are examined under applied voltages of 3.5 V and 0 V, respectively. In addition, the transmittance and absorptance properties of the two stacks are analyzed and compared for driving voltages in the range of 0–3.5 V. Finally, an experimental investigation is performed to confirm the ability of the smart window to maintain appropriate indoor illumination conditions over the course of a typical sunny day in Taiwan.

## 2. Design

**Figure 1.**
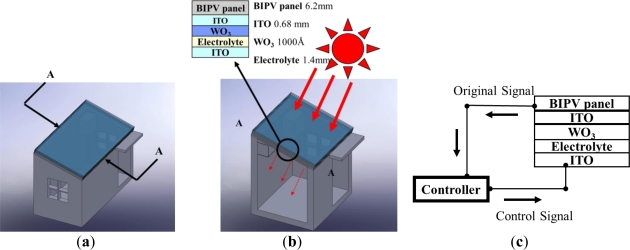
Schematic illustrations of smart window consisting of BIPV panel and electrochromic (EC) stack: (**a**) installation on roof of building; (**b**) cross-section A-A; and (**c**) signal path from BIPV module to EC stack via controller.

Figure 1 illustrates the smart window developed in this study. As shown, the window was designed for installation in the roof or dormer of a building, and comprises a BIPV panel, an EC stack and a controller. As the intensity of the solar irradiation on the BIPV panel increases, the output voltage from the BIPV module also increases. The output voltage is modulated by the controller and is then used to drive the EC stack in order to color the window.

As shown in Figure 1(b), the EC stack was sandwiched between two ITO-coated glass plates. ITO is a highly degenerated, wide-bandgap semiconductor material with relatively low resistivity and high transmittance in the visible region [25]. As a result, it is ideal for EC smart window applications. It was shown in [17] that the electrochromic effect in amorphous WO_3_ is due to the formation of tungsten bronze as a result of the double injection into the interstitial sites of the WO_3_ matrix of electrons from the active electrode and charge-compensating ions from the counter electrode. In other words, the formation of tungsten bronze occurs in accordance with the following process:

(1) $${\text{WO}}_{3}+{xM}^{+}+{xe}^{-}↹M_{x}{\text{WO}}_{3}$$

where M^+^ = H^+^, Li^+^, Na^+^ or K^+^, and e^−^ denotes an electron. Thus, when W oxide in the form of a thin transparent film acquires electrons and charge-compensating ions, it is reversibly transformed to a material with radically different properties. Specifically, the W oxide becomes absorbing if it is heavily disordered and infrared-reflecting if it is sufficiently crystalline [10].

## 3. Fabrication

**Figure 2.**
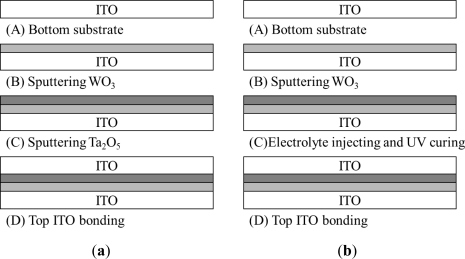
Overview of EC stack fabrication process: (**a**) ITO/WO_3_/Ta_2_O_5_/ITO and (**b**) ITO/WO_3_/lithium-polymer electrolyte/ITO.

As shown in Figure 2, two EC stacks were fabricated in the current study, namely one stack with an ITO/WO_3_/Ta_2_O_5_/ITO structure and a second stack with an ITO/WO_3_/lithium-polymer electrolyte/ITO structure.

In both cases, the stacks were fabricated using ITO-coated glass slides with a thickness of 0.68 mm and dimensions of 150 × 150 mm^2^. The WO_3_ and Ta_2_O_5_ films were deposited on the glass slides using an RF magnetron sputtering system with W and Ta_2_O_5_ targets, respectively. In performing the sputtering process, the ITO substrates were positioned at a distance of 11.4 cm from the targets (99.99% purity), the gas pressure was maintained at 0.01 torr, and the RF power was set as 200 W. The deposition process was performed using mixtures of argon (50% and 80%) and pure oxygen (50% and 20%) for the W and Ta_2_O_5_ targets, respectively, as the reactive gas. Prior to deposition, the chamber was pumped to a background pressure of 10^−6^ torr for 1 h and a pre-sputtering process was performed for 10 min in order to remove any traces of impurities from the surface of the W target [26]. RF sputtering was then performed for 1.0 h, resulting in a WO_3_ film with a thickness of approximately 1,000 Å. For comparison purposes, one stack was fabricated using a sputtered layer of Ta_2_O_5_ (thickness: 3,000 Å) (see Figure 2(a)), while the other was fabricated using a solution of LiClO_4_, propylene carbonate and UV adhesive, which was cured using UV light to form an electrolyte layer with a thickness of 1.4 mm (see Figure 2(b)). ITO glass plates were bonded to the Ta_2_O_5_ and lithium-polymer layers, respectively, to realize the finished EC stacks. Finally, a BIPV panel with a thickness of 6.2 mm was attached to the upper ITO glass plate. The BIPV panel is a crystalline light-through module (ITRI, Taiwan). Its P_MAX_, V_OC_, I_SC_, V_PMAX_ and I_PMAX_ are 1.069 W, 3.757 V, 0.412 V, 2.902 V and 0.368 A, respectively.

## 4. Results and Discussion

**Figure 3.**
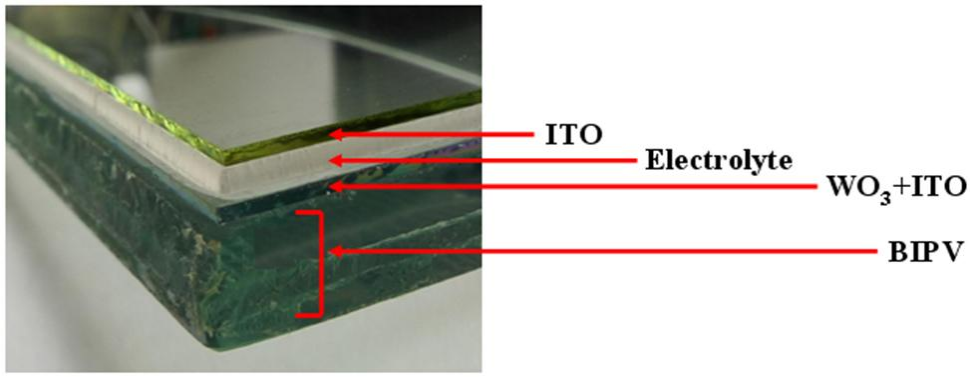
Photograph showing cross-section of smart window.

Figure 3 presents a photograph of the integrated BIPV panel/EC stack. The diffraction pattern of the WO_3_ thin-film deposited on the ITO glass slide was observed using an XRD measurement system (XRD-600 LabX, Shimadzu, Japan). The transmittance and absorptance properties of the two stacks were then investigated under various applied voltages in the range of 0–3.5 V. The illuminance of the light transmitted through the BIPV panel was measured over the period of 8:00 am to 4:00 pm on a summer day with natural light incident directly upon the panel. A controller was then designed to modulate the output voltage of the BIPV panel in such a way as to generate a suitable input voltage for the EC stack. Finally, the indoor illuminance provided by the smart window/controller under sunny daytime conditions was measured over the period of 8:00 am to 4:00 pm.

### 4.1. Diffraction Pattern of WO_3_ Thin-Film on ITO Glass Slide

**Figure 4.**
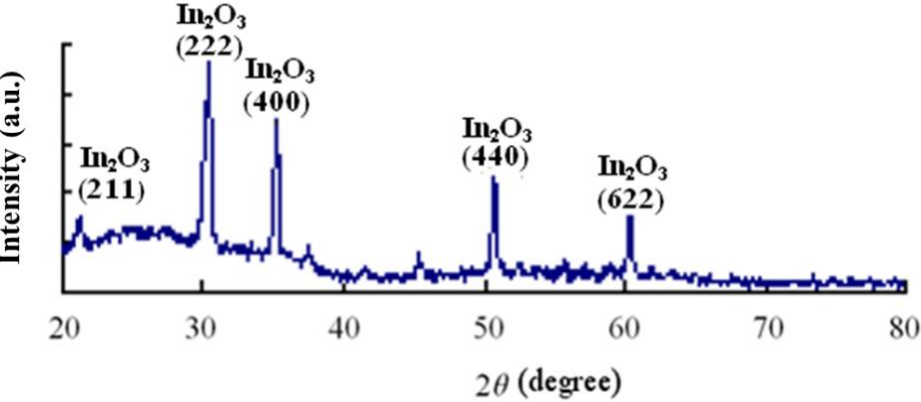
XRD diffraction pattern of deposited WO_3_ film. (Sputtering conditions: Target, W (99.99% purity); RF power, 200 W; argon flow rate, 15 sccm; oxygen flow rate, 15 sccm; working pressure, 0.01 torr).

Figure 4 shows the diffraction pattern of the WO_3_ thin-film deposited on the ITO glass slide. It is seen that apart from five distinct peaks corresponding to the ITO underlayer, the diffraction pattern is relatively featureless; indicating that the WO_3_ film has poor crystallinity.

### 4.2. Observations of Coloration and Bleaching

**Figure 5.**
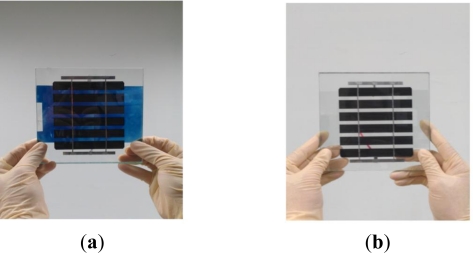
(**a**) Coloration and (**b**) bleaching of EC stack incorporated with BIPV panel at voltages of + 3.5 V and 0 V, respectively.

The coloration and bleaching behaviors of the EC stacks were observed under various voltages in the range of 0–3.5 V. It was found that for both stacks, a coloration state was induced at an applied voltage of +3.5 V in 15–20 s, while a beached state was induced at an applied voltage of 0 V in the same response time range (see Figure 5).

### 4.3. Effect of Applied Electrical Voltage on Transmittance and Absorptance Properties of EC Stacks

**Figure 6.**
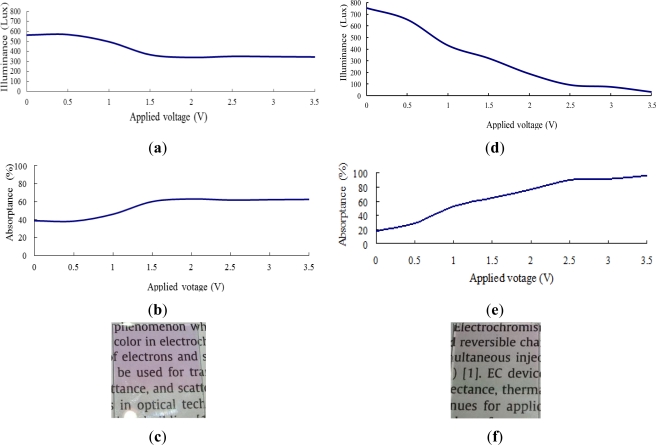
Transmittance and absorptance properties of ITO/WO_3_/Ta_2_O_5_/ITO EC stack given incident illuminance of 900 Lux. Note in (**a**–**c**), thickness of Ta_2_O_5_ film = 375 nm, while in (**d**–**f**), thickness of Ta_2_O_5_ film = 750 nm. (a) & (d): Transmittance of EC stack given applied voltage of 0–3.5 V; (b) & (e): absorptance of EC stack given applied voltage of 0–3.5 V; and (c) & (f): transparency of EC stacks given no applied voltage.

Figure 6 shows the transmittance and absorptance behaviors of the ITO/WO_3_/Ta_2_O_5_/ITO EC stack given an incident illuminance of 900 Lux and applied voltages in the range of 0–3.5 V. Note that in Figure 6(a–c), the Ta_2_O_5_ film has a thickness of 375 nm, while in Figure 6(d–f), the Ta_2_O_5_ film has a thickness of 750 nm. As shown in Figure 6(a), the indoor illuminance gradually reduces as the voltage is increased to 1.5 V. However, as the voltage is further increased to 3.5 V, the illuminance remains approximately constant at around 400 Lux. In other words, 1.5 V represents a saturation voltage, above which the number of ions transferred from the Ta_2_O_5_ layer remains approximately the same. An inspection of Figure 6(b) shows that the maximum absorptance of the EC stack is around 60%. As shown in Figure 6(d), the illuminance of the transmitted light falls rapidly with an increasing voltage due to the stronger ion movement to the WO_3_ layer when the thickness of the Ta_2_O_5_ film is increased to 750 nm. Thus, given the maximum applied voltage of 3.5 V, the illuminance of the transmitted light is less than 100 Lux. From Figure 6(e), the corresponding absorptance is found to be around 95%. Overall, the results presented in Figure 6(d,e) show that as the thickness of the Ta_2_O_5_ film is increased, the number of charge-compensating ions provided by the Ta_2_O_5_ film also increases, and thus a greater absorptance is obtained. Figure 6(c,f) illustrate the transparency of the two EC devices in the fully-bleached state given an applied voltage of 0 V. It is observed that a good transparency is obtained even given a Ta_2_O_5_ film thickness of 750 nm.

**Figure 7.**
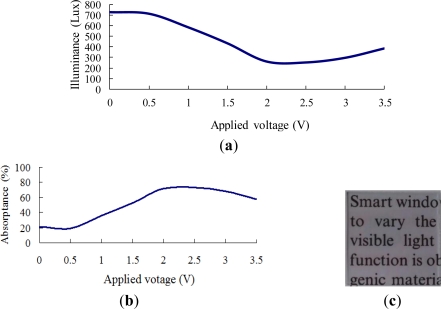
Transmittance and absorptance properties of ITO/WO_3_/lithium-polymer electrolyte/ITO stack given electrolyte thickness of 250 nm and incident illuminance of 900 Lux. (**a**) Transmittance given applied voltage of 0–3.5 V; (**b**) absorptance given applied voltage of 0–3.5 V; and (**c**) transparency of EC stack given no applied voltage.

**Figure 8.**
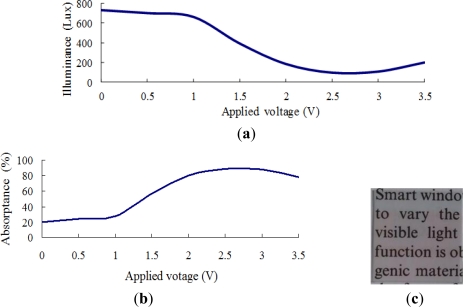
Transmittance and absorptance properties of ITO/WO_3_/lithium-polymer electrolyte/ITO stack given electrolyte thickness of 500 nm and incident illuminance of 900 Lux. (**a**) Transmittance given applied voltage of 0–3.5 V; (**b**) absorptance given applied voltage of 0–3.5 V; and (**c**) transparency of EC stack given no applied voltage.

**Figure 9.**
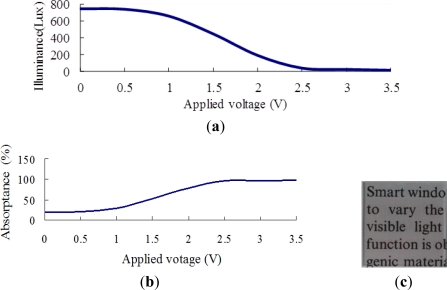
Transmittance and absorptance properties of ITO/WO_3_/lithium-polymer electrolyte/ITO stack given electrolyte thickness of 750 nm and incident illuminance of 900 Lux. (**a**) Transmittance given applied voltage of 0–3.5 V; (**b**) absorptance given applied voltage of 0–3.5 V; and (**c**) transparency of EC stack given no applied voltage.

**Figure 10.**
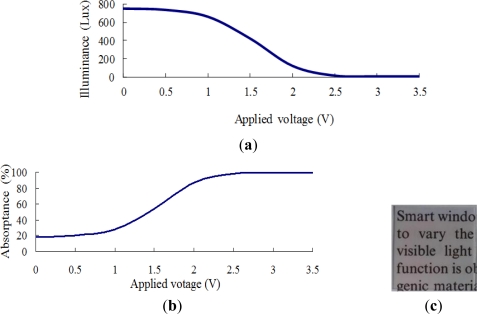
Transmittance and absorptance properties of ITO/WO_3_/lithium-polymer electrolyte/ITO stack given electrolyte thickness of 1,000 nm and incident illuminance of 900 Lux. (**a**) Transmittance given applied voltage of 0–3.5 V; (**b**) absorptance given applied voltage of 0–3.5 V; and (**c**) transparency of EC stack given no applied voltage.

Figures 7–10 show the transmittance and absorptance behaviors of the ITO/WO_3_/lithium-polymer electrolyte/ITO stack given applied voltages of 0–3.5 V and electrolyte film thicknesses of 250, 500, 750 and 1,000 nm. Note that the illuminance of the incident light is 900 Lux in all cases. Figure 7(a) shows that the illuminance of the transmitted light decreases as the applied voltage is increased to 2 V, but then increases slightly as the voltage is increased further due to an insufficient supply of charge-compensating ions from the thin (250 nm) electrolyte film. A similar tendency is observed in the EC stack with an electrolyte thickness of 500 nm (Figure 8(a)). However, for the EC stacks with an electrolyte thickness greater than 500 nm, the illuminance of the transmitted light saturates at an applied voltage of approximately 2.5 V (Figures 9(a) and 10(a)). The corresponding absorptance is found to be approximately 99% (Figures 9(b) and 10(b)). Figures 7(c), 8(c), 9(c) and 10(c) illustrate the transparency properties of the four EC stacks. It is seen that a good transparency is maintained even for an electrolyte layer with a thickness of 1000 nm.

Comparing Figures 6 and 7–10, it is seen that the EC stack with a lithium-polymer electrolyte yields a better absorptance performance under the maximum applied voltage (approximately 99%) than the EC stack with a Ta_2_O_5_ film (95%) due to the difference of their ion conductivity. Thus, the ITO/WO_3_//lithium-polymer electrolyte/ITO stack with an electrolyte thickness of 1,000 nm was chosen for integration with the BIPV panel to form the BIPV-powered smart window.

### 4.4. Modulation of Indoor Illuminance

**Figure 11.**
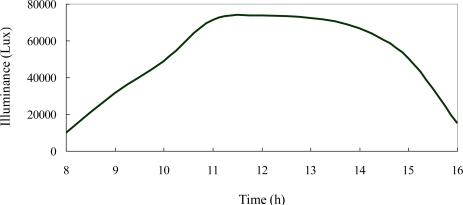
Variation in illuminance of light incident on BIPV panel between 8:00 am and 4:00 pm during a typical summer day.

Figure 11 shows the variation in the illuminance of the light incident on the BIPV panel over the period of 8:00 am to 4:00 pm on a typical summer day in Taiwan with the natural light incident directly on the smart window. It is seen that an illuminance of more than 70,000 Lux is obtained at midday. This value is far higher than the recommended indoor illuminance range of 750–1,500 Lux during daytime hours [27]. Thus, a significant reduction in the indoor illuminance is required.

**Figure 12.**
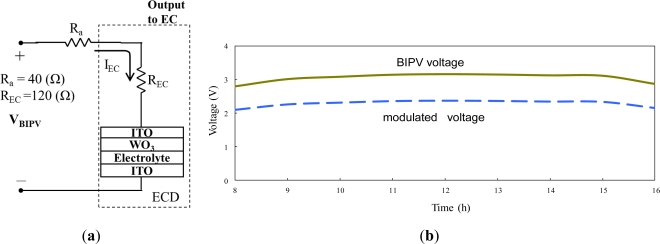
(**a**) Layout of divider circuit in voltage controller, and (**b**) variations of original and modulated output voltages from BIPV panel between 8:00 am and 4:00 pm during a typical summer day.

To optimize the indoor illuminance during the daytime hours, it is necessary to modulate the absorptance of the EC stack in accordance with changes in the intensity of the solar irradiation on the BIPV panel of the smart window. The results presented in Section 4.3 have shown that the absorptance of the ITO/WO_3_/lithium-polymer electrolyte/ITO stack increases with an increasing driving voltage. Meanwhile, the results presented in Section 4.4 have shown that the illuminance of the light transmitted through the BIPV module increases as the intensity of the solar irradiation increases. In other words, to maintain an acceptable indoor illuminance condition, the absorptance of the EC stack should be increased as the outdoor illuminance increases. For a photovoltaic device such as a BIPV panel, the output voltage increases as the illuminance of the incident light on the panel increases. Thus, in the present study, the output voltage from the BIPV panel is modulated adaptively in order to generate the EC driving voltage required to maintain the optimal indoor illuminance conditions given changes in the intensity of the outdoor solar irradiation. Figure 12(a) illustrates the layout of the control circuit used to modulate the BIPV output voltage. The ITO/WO_3_/lithium-polymer electrolyte/ITO EC stack has an operating current of 20 mA and an internal resistance of 120 Ω. Thus, the controller was implemented using a series connection of one 40 Ω of resistor; causing the output voltage of the BIPV panel to be divided with a 75% ratio for optimizing the input voltage of the EC device. Figure 12(b) shows the variations of the original and modulated voltages from the BIPV module over the period of 8:00 am to 4:00 pm. The modulated voltage can control the absorptance effect with an optimization ratio following the experimental results of Figure 10(b).

**Figure 13.**
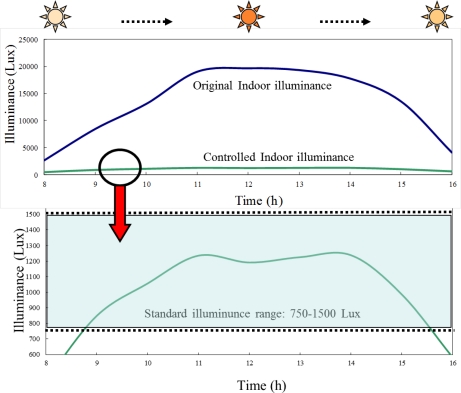
Variations of original indoor illuminance (**upper**) and controlled indoor illuminance (**lower**) between 8:00 am and 4:00 pm during a typical summer day.

Figure 13 shows the variation of the original transmitted sunlight intensity through the smart window over the period of 8:00 am to 4:00 pm (Figure 13(upper)) and the corresponding variation of the indoor illuminance, as controlled by the smart window (Figure 13(lower)). It can be seen that the smart window ensures that the indoor illuminance remains within the recommended range of 750–1,500 Lux from 8.40 to 15.40 despite significant changes in the outdoor solar irradiation intensity.

## 5. Conclusions

This study has fabricated and characterized a BIPV-powered smart window comprising a BIPV panel and an EC stack for optimizing the indoor illuminance conditions without the need for shading devices. The novelty of the study is that the output voltage of the BIPV panel varies in accordance with the intensity of the incident light and can be modulated in such a way to generate the EC stack voltage required to maintain the indoor illuminance within a specified range without any requirement of external energy source. Two all-solid-state EC stacks have been fabricated, namely one stack with an ITO/WO_3_/Ta_2_O_5_/ITO structure and one stack with an ITO/WO_3_/lithium-polymer electrolyte/ITO structure. The experimental results have shown that the latter stack yields a larger absorptance (approximately 99%) given the maximum driving voltage of 3.5 V. A control scheme has been proposed for modifying the output voltage of the BIPV panel in such a way as to optimize the absorptance of the ITO/WO_3_/lithium-polymer electrolyte/ITO stack in response to changes in the outdoor solar illuminance. Though ITO is an ideal solution for the utilization of the EC-based smart windows, the scarcity of its main component (In) should be noticed and its replacement component is hoped to be found in the field of materials science and engineering for the universalization of the EC-based smart windows. In the paper, it has been shown that the smart window ensures that the indoor illuminance remains within the recommended range of 750–1,500 Lux despite significant changes in the intensity of the outdoor solar irradiation.
